# Clinical impact of tricuspid regurgitation in patients with acute myocardial infarction

**DOI:** 10.1002/ehf2.15375

**Published:** 2025-07-14

**Authors:** Shun Nishino, Chiharu Nishino, Michikazu Nakai, Kensaku Nishihira, Nehiro Kuriyama, Yoshisato Shibata

**Affiliations:** ^1^ Department of Cardiology Miyazaki Medical Association Hospital Cardiovascular Center Miyazaki Japan; ^2^ Clinical Research Support Center University of Miyazaki Hospital Miyazaki Japan

**Keywords:** Echocardiography, Myocardial infarction, Prognosis, Three‐dimensional echocardiography, Tricuspid regurgitation, Valvular heart disease

## Abstract

**Aims:**

The clinical impact of tricuspid regurgitation (TR) in patients after acute myocardial infarction (AMI) is largely unknown. The aim of this study was to clarify the prevalence and prognostic impact of TR in post‐AMI patients treated with appropriate primary percutaneous coronary intervention (PCI).

**Methods and results:**

Three hundred fifty‐one consecutive patients with first‐onset AMI who underwent successful primary PCI from July 2014 to December 2018 were retrospectively examined. Standard two‐ and three‐dimensional echocardiography were performed at discharge. Based on the presence or absence of mild or greater TR, patients were divided into TR (+) and TR (−) groups, respectively. The primary outcome was the incidence of major adverse cardiac events (MACE), defined as the composite of death, re‐hospitalization for congestive heart failure and recurrent MI. Seventy‐eight (22.2%) patients had mild or greater TR. Kaplan–Meier analysis showed that the cumulative 6‐year incidence of MACE was significantly higher in the TR (+) group (hazard ratio, 2.56 [95% confidence interval, 1.48–4.44]; *P* < 0.001). In the analysis of the severity of TR, the prognosis of patients with mild TR was significantly worse than that of patients without TR (*P* = 0.026). Multivariable analysis identified the left anterior descending coronary artery as the culprit vessel, left atrial dilation (>34 mL/m^2^), reduced left ventricular ejection fraction (<50%) and the presence of significant (≥mild) ischaemic mitral regurgitation as independent predictors of mild or greater residual TR after primary PCI for AMI at discharge. Following adjustment for significant clinical parameters, mild or greater TR at discharge was still associated with a significant hazard ratio for the occurrence of MACE (1.87, [95% confidence interval, 1.01–3.48]; *P* = 0.048).

**Conclusions:**

The presence of mild or greater TR at discharge may serve as a poor prognostic marker in patients with first‐onset AMI. In addition to traditional clinical risk factors, it is important to pay more attention to TR and to manage it appropriately.

## Graphical abstract

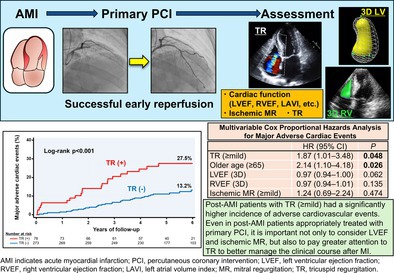

## Introduction

The prognosis after acute myocardial infarction (AMI) may sometimes be poor, despite improvements associated with early reperfusion with primary percutaneous coronary intervention (PCI), the development of pharmacological therapy and the identification of residual risk.^1^, ^2^, ^3^, ^4^ Many studies have reported prognostic factors in patients after AMI, such as left ventricular ejection fraction (LVEF)^5^, ^6^ and ischaemic mitral regurgitation (MR).^7^ Tricuspid regurgitation (TR) in patients with heart failure, irrespective of LVEF, has been found to worsen prognosis.^8^, ^9^, ^10^, ^11^ Furthermore, poor prognosis has been reported in patients with mild or even moderate functional TR.^12^ Mild or greater TR is considered to be a late‐stage marker of poor outcome^10^, ^12^, ^13^, ^14^ and worse prognosis in patients with left‐sided valvular disease such as MR^15^, ^16^, ^17^ and aortic stenosis.^15^ The recent availability of percutaneous therapeutic interventions for TR has emphasized its clinical significance.^18^, ^19^ Few studies have focused on TR in patients after AMI,^20^ as it is often not seen as related to the left heart system. Although many studies have examined the characteristics and prognostic impact of left heart valvular disease in post‐AMI patients, few have analysed TR, and its clinical significance is not yet fully understood. This is the first study to focus on the prevalence and prognostic impact of TR in patients with AMI who have undergone appropriate reperfusion therapy, using accurate three‐dimensional transthoracic echocardiographic (TTE) assessment.

## Methods

### Study population

This single‐centre, observational study was performed at the Miyazaki Medical Association Hospital, a cardiovascular centre with a 24/7 primary PCI service. Of 750 consecutive patients with first‐onset AMI who were transported to our hospital from July 2014 to December 2018, 351 who underwent successful emergency PCI and had adequate three‐dimensional TTE assessment performed at discharge were retrospectively enrolled (*Figure* *S1*). The diagnosis of AMI was established according to the Fourth Universal Definition of Myocardial Infarction, based on symptoms, diagnostic electrocardiographic changes, echocardiographic findings and elevation of myocardial biomarkers.^21^ Exclusion criteria were as follows: (1) coexisting cardiomyopathy or structural heart disease such as primary valve disease and congenital heart disease, (2) cardiogenic shock or mechanical complications of AMI (papillary muscle rupture, ventricular septal rupture and free wall rupture) on arrival and (3) rhythm disorder complications such as atrial fibrillation/flutter or atrioventricular block at the time of echocardiography. This study protocol was approved by the institutional review board and ethics committee of Miyazaki Medical Association Hospital (#2021‐43). All patients provided informed written consent.

### Echocardiographic assessment

**Figure 1:**
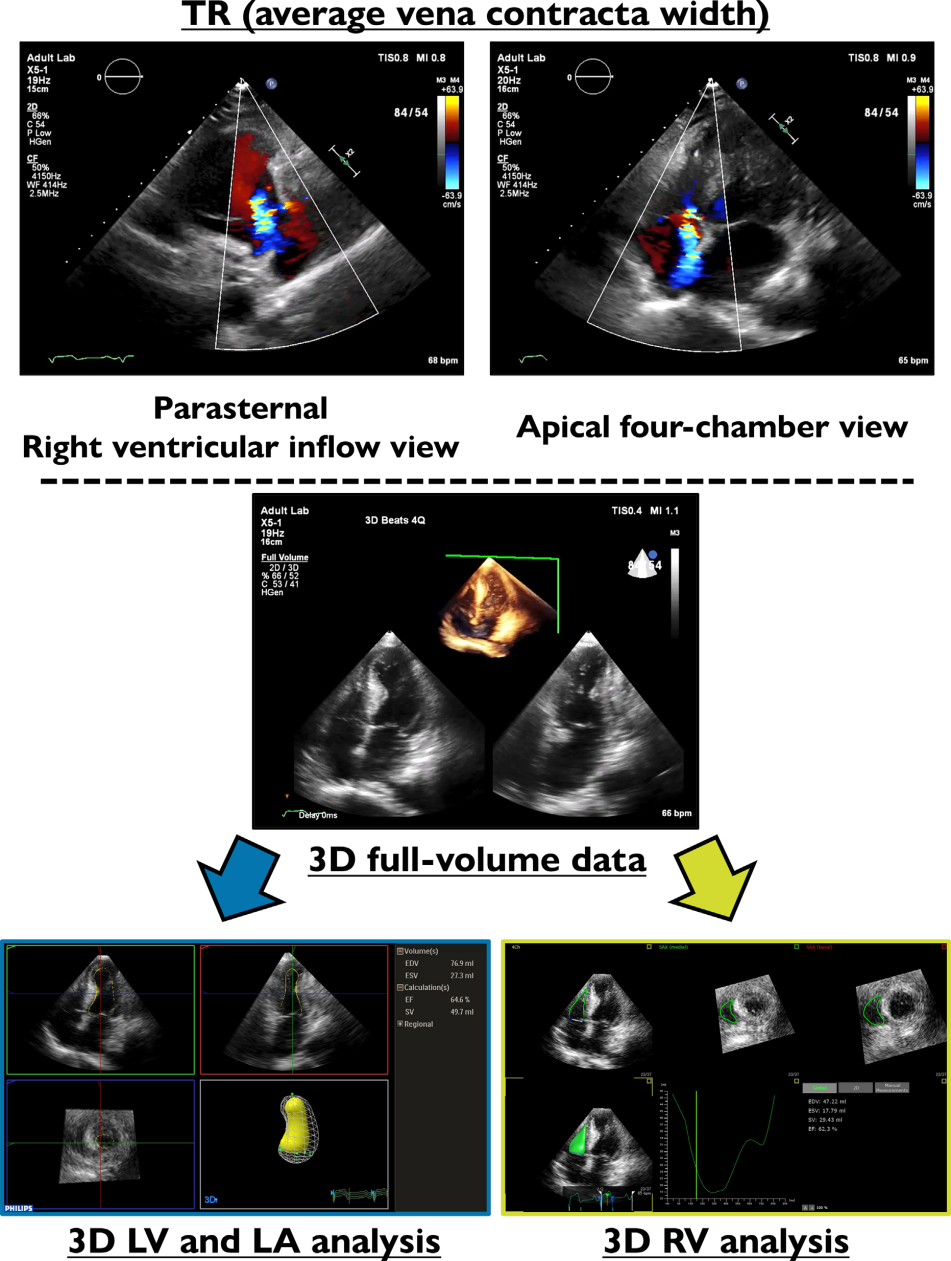
The severity of tricuspid regurgitation was semi‐quantified by the average vena contracta width on parasternal right ventricular inflow and apical four‐chamber views with colour Doppler images. Three‐dimensional full‐volume data were transferred to personal computers for offline analysis. QLAB15 3D Quantification Advanced software was used for left ventricular and left atrial volume analysis. 4D RV‐Function 2.0 software was used for right ventricular volume analysis. LA, left atrium; LV, left ventricle; RV, right ventricle; TR, tricuspid regurgitation; 3D, three‐dimensional.

Each patient underwent standard two‐dimensional and Doppler echocardiography, followed by real‐time three‐dimensional TTE at discharge [9 (8–11) days after primary PCI] (*Figure* *1*). TTE was performed using IE33, EPIQ7 and EPIQ CVX systems equipped with an X‐5 probe (Philips Medical Systems, Andover, MA, USA). The two‐ and three‐dimensional TTE datasets were digitally stored and transferred to computers for offline analysis. All measurements and analysis were independently performed by two experienced observers (S.N. and C.N.) blinded to the clinical data.

### Two‐dimensional echocardiographic study

Left ventricular (LV) end‐diastolic diameter and LV end‐systolic diameter were measured on parasternal long‐axis images. TR was carefully evaluated by two‐dimensional colour Doppler echocardiography. The degree of TR was semi‐quantified by colour Doppler imaging, based on the average vena contracta width measured from two different views: the parasternal right ventricular inflow view and the apical four‐chamber view. The final value was calculated by averaging the measurements obtained from these two views to improve accuracy, as recommended by the American Society of Echocardiography.^22^ TR severity was determined by the average vena contracta width as follows: mild, <3 mm; moderate, ≥3, <7 mm; severe, ≥7 mm. Similarly, MR was semi‐quantified on apical two‐ and four‐chamber colour Doppler images.^7^, ^23^, ^24^, ^25^ MR severity was determined by the average vena contracta width as follows: mild, ≥3, <5 mm; moderate, ≥5, <8 mm; severe, ≥8 mm. Offline measurements of echocardiographic parameters were performed using TomTec‐Arena (TomTec Imaging Systems, Munich, Germany).

### Three‐dimensional echocardiographic study

Using a real‐time three‐dimensional echocardiographic system, we obtained transthoracic volumetric images (full‐volume mode) in the apical view. The volumetric frame rate was 15–25 frames/s, with an imaging depth of 12–19 cm. Before acquiring the full‐volume image, we carefully located the transducer position at the apex in biplane mode, with a breath hold when possible. All volumetric images were transferred to personal computers for offline analysis using QLAB15 3D Quantification Advanced software (Philips Medical Systems) and 4D RV‐Function 2.0 software (TomTec Imaging Systems). Structural and functional parameters of the left atrium, left ventricle and right ventricle (RV) were assessed using the appropriate software. Left atrial volume was categorized using 34 mL/m^2^ as the cutoff, in accordance with the definition of left atrial dilation. LVEF was classified as preserved (≥50%) or reduced (<50%) function. Right ventricular ejection fraction (RVEF) was classified as normal (≥45%) or reduced (<45%).^26^, ^27^, ^28^

### Endpoints and definitions

Prognostic information after hospital discharge was obtained from hospital medical records or by contacting patients or family members for up to 6 years [1924 (1609–2359) days]. The primary outcome of this study was the incidence of major adverse cardiac events (MACE), defined as the composite of all‐cause death, re‐hospitalization for congestive heart failure and recurrent MI.

### Statistical analysis

This study aimed to evaluate risk factors for TR after primary PCI for AMI, and its impact on MACE. Patients with or without mild or greater TR were also compared. Continuous variables are expressed as the mean ± standard deviation or the median and interquartile range, as appropriate. Categorical variables are reported as numbers and frequency (%). Comparisons of each parameter were performed using the *t*‐test or Mann–Whitney *U* test, as appropriate. Categorical data were compared using the likelihood‐ratio chi‐squared test or Fisher's exact test, as appropriate. Kaplan–Meier analysis was used to estimate the cumulative incidence of outcomes at 6 years, and the log‐rank test was used to assess between‐group differences. Pairwise comparisons were additionally performed using Dunnett's test to account for multiple testing. Univariable and multivariable logistic regression analyses of clinically important factors were performed to evaluate predictors of mild or greater TR at discharge. Propensity scores matching was conducted to consider the potential impact of sex differences using the 1:1 nearest‐neighbour matching algorithm without replacement. A calliper was set a width of 0.25 standard deviations of the logit of the propensity score. This matching process aimed to create a comparable cohort to assess the independent association between sex and TR while reducing confounding effects. In addition, univariable analysis of MACE was performed using covariates consisting of clinically important factors, and multivariable Cox proportional hazards analyses were conducted on the factors with significant differences. All analyses were performed using STATA 18 (StataCorp LLC, College Station, TX, USA). A two‐sided *P* value of <0.05 indicated significance in all tests.

To evaluate reproducibility, coefficients of variations were used to evaluate inter‐ and intra‐observer variabilities in 10 randomly selected cases. When assessing inter‐observer variability, measurements were repeated by a second independent observer blinded to the first observer's results. Intra‐observer variability was examined by comparing each observer's analyses on the same 10 cases, separated by at least 3 months.

## Results

We serially examined 351 patients with AMI at the time of hospital discharge. TR was absent or trivial in 273 (77.8%) of these patients, mild in 63 (17.9%) and moderate in 15 (4.3%). No patients had severe TR. Patients' baseline characteristics are summarized in *Table* *1*. The mean age was 66 ± 12 years, 271 (77.2%) patients were men, and 291 (82.9%) had ST‐segment elevation MI. All patients underwent successful primary PCI for their single culprit lesion. The average door‐to‐balloon time was 65 (53–85) minutes, and the average onset‐to‐reperfusion time was 249 (160–485) minutes. The median hospitalization duration was 14 (12–18) days. TTE assessment at discharge was performed 9 (8–11) days after primary PCI. The median LVEF at discharge was 55.2 (48.8–59.6) %. Mild or greater TR was present in 78 patients (22.2%). Based on the presence or absence of mild or greater TR, patients were divided into TR (+) and TR (−) groups, respectively.

**Table 1:** Patients' baseline characteristics

	Total	TR (+)	TR (−)	*P*
	(*n* = 351)	(*n* = 78)	(*n* = 273)	
Age, years	66 ± 12	71 ± 12	65 ± 12	<0.001
Male	271 (77.2%)	53 (67.9%)	218 (79.9%)	0.027
BMI, kg/m^2^	24.3 ± 3.6	22.2 ± 3.2	24.8 ± 3.6	<0.001
BSA, m^2^	1.69 ± 0.20	1.60 ± 0.20	1.72 ± 0.19	<0.001
Cardiovascular risk factors				
Hypertension	224 (63.8%)	53 (67.9%)	171 (62.6%)	0.389
Diabetes mellitus	92 (26.2%)	15 (19.2%)	77 (28.2%)	0.112
Dyslipidaemia	225 (64.1%)	47 (60.3%)	178 (65.2%)	0.422
Hyperuricemia	19 (5.4%)	6 (7.7%)	13 (4.8%)	0.313
Smoking history	201 (57.3%)	44 (56.4%)	157 (57.5%)	0.863
Renal function; eGFR, mL/min/1.73 m^2^	71.0 (58.2–84.1)	71.8 (55.9–83.1)	70.9 (59.2–84.4)	0.546
Family history	60 (17.1%)	12 (15.4%)	48 (17.6%)	0.649
Known ischaemic heart disease	3 (0.9%)	1 (1.3%)	2 (0.7%)	0.642
Paroxysmal atrial fibrillation	6 (1.7%)	3 (3.8%)	3 (1.1%)	0.099
Presentation at admission				
ST‐segment elevation myocardial infarction	291 (82.9%)	67 (85.9%)	224 (82.1%)	0.426
Killip class I	315 (89.7%)	65 (83.3%)	250 (91.6%)	0.034
BNP, pg/mL	37.1 (15.4–97.2)	71.1 (25.3–161.5)	34.2 (13.7–83.2)	<0.001
Angiographic and procedural characteristics				
Culprit vessel				
LAD	197 (56.1%)	50 (64.1%)	147 (53.8%)	0.110
RCA	101 (28.8%)	12 (15.4%)	89 (32.6%)	0.003
LCX	47 (13.4%)	14 (17.9%)	33 (12.1%)	0.180
LMT	6 (1.7%)	2 (2.6%)	4 (1.5%)	0.510
Multivessel disease	110 (31.3%)	26 (33.3%)	84 (30.8%)	0.667
Door‐to‐balloon time, min	65 (53–85)	69 (51–90)	65 (55–80)	0.326
Onset‐to‐reperfusion time, min	249 (160–485)	258 (175–680)	240 (154–435)	0.090
Stent use	336 (95.7%)	73 (93.6%)	263 (96.3%)	0.290
Peak CK, U/L	2,050 (912–3,570)	2,320 (967–4,205)	2,005 (873–3,460)	0.152
Peak CK‐MB, U/L	191 (88–327)	225 (82–380)	174 (89–310)	0.094
Hospital stay, days	14 (12–18)	16 (14–21)	14 (12–17)	<0.001
Medications (before the onset)				
Beta‐blocker	4 (1.1%)	0 (0.0%)	4 (1.5%)	0.364
ACE‐I or ARB	72 (20.5%)	20 (25.6%)	52 (19.0%)	0.203
MRA	1 (0.3%)	0 (0.0%)	1 (0.4%)	0.778
Loop diuretic	3 (0.9%)	1 (1.3%)	2 (0.7%)	0.531
SGLT2 inhibitors	4 (1.1%)	0 (0.0%)	4 (1.5%)	0.364

Data are expressed as mean ± standard deviation, median (interquartile range) or number (%), as appropriate.

ACE‐I, angiotensin‐converting enzyme inhibitor; ARB, angiotensin II receptor blocker; BMI, body mass index; BNP, B‐type natriuretic peptide; BSA, body surface area; CK, creatine kinase; CK‐MB, creatine kinase‐myocardial band; eGFR, estimated glomerular filtration rate; LAD, left anterior descending coronary artery; LCX, left circumflex coronary artery; LMT, left main trunk; MRA, mineralocorticoid receptor antagonist; RCA, right coronary artery; SGLT2, sodium glucose cotransporter 2; TR, tricuspid regurgitation.

### Patients' baseline clinical characteristics

Compared to the TR (−) group, the TR (+) group had significantly higher percentages of patients who were elderly, female or had a low BMI. There were no significant differences in cardiovascular risk factors between the two groups. On admission, there was no significant difference in the percentage of patients with ST‐segment elevation MI, but the percentage with inferior MI was significantly lower in the TR (+) group than in the TR (−) group. The B‐type natriuretic peptide level was significantly higher in the TR (+) group, but it was not above the clinically significant limit (>100 pg/mL).

### Baseline angiographic characteristics and procedural outcomes

The proportion of cases with the right coronary artery as the culprit lesion was significantly higher in the TR (−) group than in the TR (+) group. There were no significant between‐group differences in door‐to‐balloon time, onset‐to‐reperfusion time, peak creatine kinase level, creatine kinase‐myocardial band level, or the proportions of stent use or multivessel disease. Hospitalization duration was longer in the TR (+) group than in the TR (−) group. At the time of admission, there was no significant difference in the prescription rate of heart failure medications, including diuretics, between the two groups. However, at evaluation, the TR (+) group was significantly more likely to receive diuretics than the TR (−) group (*Table* *S1*).

### Baseline echocardiographic parameters

Baseline echocardiographic parameters for cardiac chambers and functional atrioventricular valve regurgitation are shown in *Table* *2*. The TR (+) group had a significantly greater left atrial volume, LV diameter, and LV volume than the TR (−) group, but a lower LVEF. RV end‐diastolic diameter and volume were significantly greater in the TR (+) group, but there were no significant differences in RVEF, fractional area change, or free wall right ventricular strain parameter. Pulmonary artery systolic pressure was significantly higher in the TR (+) group. Significant (≥mild) ischaemic MR was significantly more common in the TR (+) group than the TR (−) group.

**Table 2:** Baseline echocardiographic parameters

	Total	TR (+)	TR (−)	*P*
	(*n* = 351)	(*n* = 78)	(*n* = 273)	
Left atrial and ventricular parameters				
LA volume, mL/m^2^	25.0 (19.2–32.5)	30.1 (22.3–37.7)	23.6 (18.7–30.2)	<0.001
LA dilation (>34 mL/m^2^)	68 (19.4%)	32 (41.0%)	36 (13.2%)	<0.001
LV end‐diastolic diameter, mm/m^2^	28.6 (26.1–31.1)	30.2 (28.3–32.9)	28.0 (26.0–30.6)	<0.001
LV end‐systolic diameter, mm/m^2^	19.5 (17.3–22.4)	21.1 (18.2–24.1)	19.1 (17.0–21.8)	<0.001
LV end‐diastolic volume, mL/m^2^	45.9 (37.8–53.6)	49.0 (40.2–60.0)	45.2 (37.0–52.8)	0.007
LV end‐systolic volume, mL/m^2^	20.4 (15.6–26.8)	22.9 (18.4–33.5)	19.8 (15.4–25.1)	<0.001
LV ejection fraction (3D), %	55.2 (48.8–59.6)	52.0 (41.9–57.8)	55.7 (50.2–59.9)	<0.001
Reduced LV ejection fraction (<50%)	100 (28.5%)	34 (43.6%)	66 (24.2%)	<0.001
Right ventricular parameters				
RV end‐diastolic volume, mL/m^2^	35.4 (30.6–40.2)	36.6 (32.0–43.2)	35.0 (30.2–39.6)	0.031
RV end‐systolic volume, mL/m^2^	14.7 (12.3–17.7)	15.3 (13.3–18.2)	14.4 (12.0–17.5)	0.071
RV ejection fraction (3D), %	58.0 (53.2–62.7)	57.6 (51.9–62.7)	58.2 (53.4–62.6)	0.830
Reduced RV ejection fraction (<45%)	15 (4.3%)	3 (3.8%)	12 (4.4%)	0.847
RV basal end‐diastolic diameter, mm/m^2^	18.2 (16.1–19.8)	19.8 (18.0–22.1)	17.7 (15.7–19.2)	<0.001
RV mid end‐diastolic diameter, mm/m^2^	15.4 (13.6–17.7)	16.4 (14.5–18.9)	15.0 (13.3–17.2)	<0.001
FAC, %	52.8 (46.1–56.5)	52.7 (45.1–56.6)	52.8 (46.1–56.4)	0.769
RVLS (free wall), %	29.0 (25.3–33.0)	29.5 (26.7–33.5)	28.8 (25.1–33.0)	0.152
Pulmonary artery systolic pressure, mmHg	24.4 (21.0–28.0)	28.0 (25.0–33.0)	23.0 (20.0–26.0)	<0.001
Valvular disease				
Degree of TR				
No or trivial	273 (77.8%)	0 (0.0%)	273 (100.0%)	
Mild	63 (17.9%)	63 (80.8%)	0 (0.0%)	
Moderate	15 (4.3%)	15 (19.2%)	0 (0.0%)	
Severe	0 (0.0%)	0 (0.0%)	0 (0.0%)	
Degree of ischaemic MR				
No or trivial	233 (66.4%)	37 (47.4%)	196 (71.8%)	
Mild	84 (23.9%)	25 (32.1%)	59 (21.6%)	
Moderate	32 (9.1%)	14 (17.9%)	18 (6.6%)	
Severe	2(0.6%)	2 (2.6%)	0 (0.0%)	

Data are expressed as median (interquartile range) or n (%) of patients.

3D, three‐dimensional; FAC, fractional area change; LA, left atrial; LV, left ventricular; MR, mitral regurgitation; RV, right ventricular; RVLS, right ventricular longitudinal strain; TR, tricuspid regurgitation; VC, vena contracta.

### Predictive factors of mild or greater TR at discharge

Multivariable analysis identified the left anterior descending coronary artery as the culprit vessel, left atrial dilation (>34 mL/m^2^), reduced LVEF (<50%), and the presence of significant (≥mild) ischaemic MR as independent predictors of mild or greater residual TR after primary PCI for AMI at discharge (*Table* *3*). In contrast, older age (≥65 years old), male sex and reduced RVEF (<45%) were not significantly associated with residual TR. Age was categorized using 65 years as the cutoff, in accordance with commonly accepted definitions of elderly patients in clinical settings. Considering that the overall cohort was predominantly male, we performed propensity score matching analysis to balance for sex, and the results demonstrated that the same factors identified in the overall cohort remained as predictors of mild or greater TR (*Table* *3*). This suggests that the associations observed in the entire study population were not solely driven by sex differences.

**Table 3:** Predictive factors for mild or greater TR after primary PCI for first‐onset AMI

Overall cohort	Univariable		Multivariable	
(*n* = 351)	OR (95% CI)	*P*	OR (95% CI)	*P*
Older age (≥65 years old)	2.60 (1.51–4.50)	0.001	1.82 (0.97–3.38)	0.060
Male	0.53 (0.31–0.94)	0.029	0.73 (0.38–1.40)	0.339
Culprit vessel (LAD)	1.53 (0.91–2.58)	0.109	2.03 (1.10–3.74)	0.024
LA dilation (>34 mL/m^2^)	4.58 (2.59–8.11)	<0.001	3.28 (1.68–6.41)	0.001
Reduced LVEF (<50%)	2.42 (1.43–4.10)	0.001	1.98 (1.09–3.59)	0.024
Reduced RVEF (<45%)	0.88 (0.24–3.21)	0.847	0.51 (0.12–2.15)	0.361
Significant ischaemic MR (≥mild)	2.82 (1.68–4.73)	<0.001	1.90 (1.04–3.49)	0.037

Sex adjusted propensity score match	Univariable		Multivariable	
(*n* = 160)	OR (95% CI)	*P*	OR (95% CI)	*P*
Older age (≥65 years old)	2.24 (1.05–4.78)	0.037	1.95 (0.76–4.97)	0.164
Culprit vessel (LAD)	2.49 (1.17–5.31)	0.018	4.02 (1.53–10.58)	0.005
LA dilation (>34 mL/m^2^)	3.77 (1.77–8.05)	0.018	2.99 (1.10–8.07)	0.031
Reduced LVEF (<50%)	3.16 (1.52–6.59)	0.002	3.04 (1.29–7.20)	0.011
Reduced RVEF (<45%)	1.37 (0.33–5.75)	0.668	0.96 (0.18–5.11)	0.961
Significant ischaemic MR (≥mild)	3.61 (1.75–7.45)	0.001	2.46 (1.03–5.87)	0.043

AMI, acute myocardial infarction; CI, confidence interval; LA, left atrial; LAD, left anterior descending coronary artery; LVEF, left ventricular ejection fraction; MR, mitral regurgitation; OR, odds ratio; PCI, percutaneous coronary intervention; RVEF, right ventricular ejection fraction; TR, tricuspid regurgitation.

### Impact on cardiac events of mild or greater TR after primary PCI for AMI

**Figure 2:**
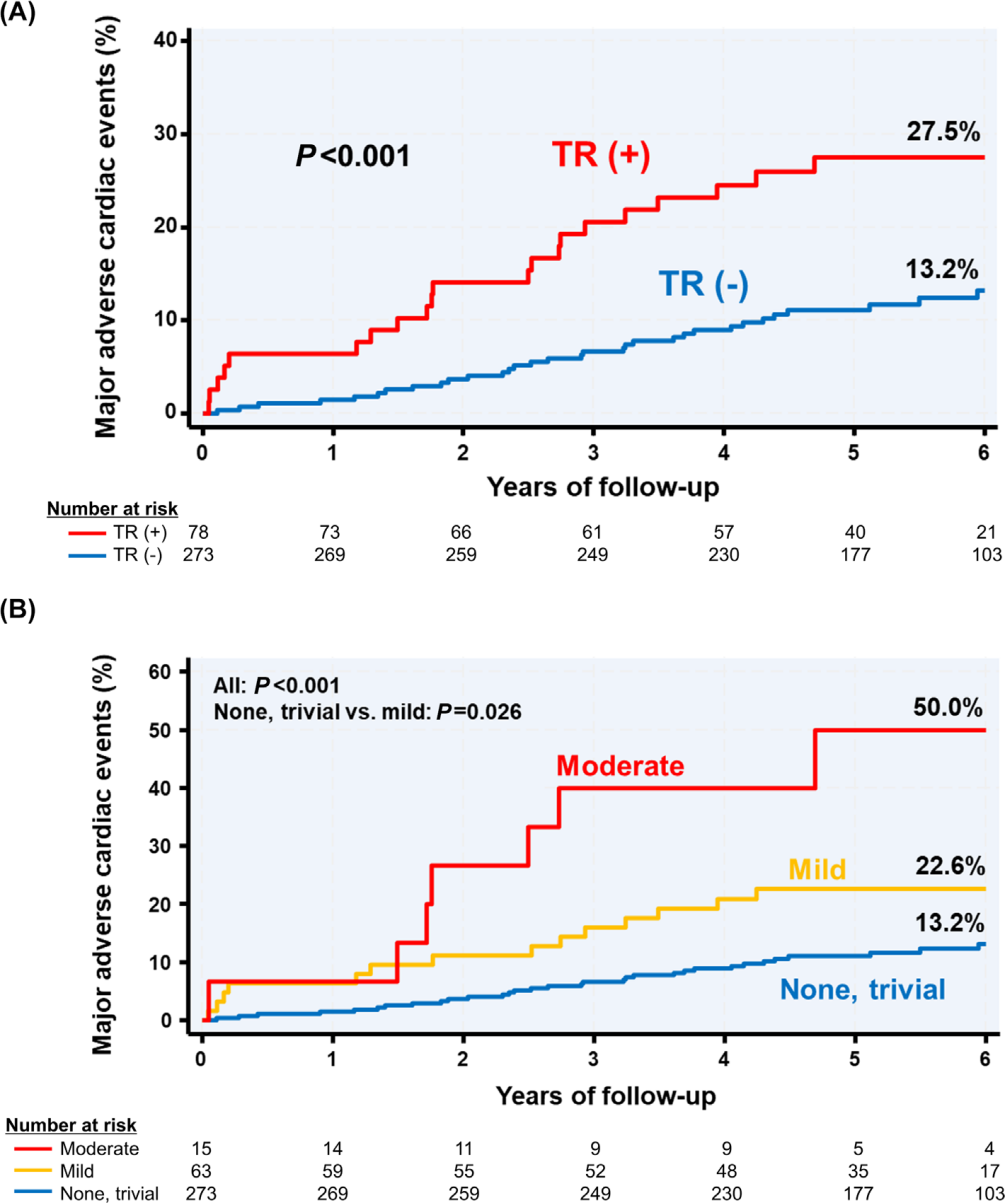
At 6 years, the rate of major adverse cardiac events (the composite of all‐cause death, re‐hospitalization for congestive heart failure, and recurrent myocardial infarction) was higher in patients with mild or greater tricuspid regurgitation at discharge compared with those without (27.5% vs. 13.2%, respectively; log‐rank *P* < 0.001) (A). In the analysis of the severity of TR, the prognosis of patients with mild TR was significantly worse than that of patients without TR (B). TR, tricuspid regurgitation.

Over a median period of 5.1 (4.3–6.0) years, 53 (15.1%) patients in the entire cohort had adverse cardiac events. The cumulative 6‐year incidence rates of MACE were 27.5% in the TR (+) and 13.2% in the TR (−) group. The breakdown of MACE events for each group is summarized in the table (*Table* *S2*). The median observation duration did not differ significantly between the two groups (*P* = 0.25). Kaplan–Meier analysis showed that the cumulative 6‐year incidence of MACE was significantly lower in the TR (−) group than in the TR (+) group (log‐rank *P* < 0.001) (*Figure* *2* *A*). In the analysis of the severity of TR, the prognosis of patients with mild TR was significantly worse than that of patients without TR (*P* = 0.026, Dunnett's test) (*Figure* *2* *B*). In the univariate Cox proportional hazards analysis using the no or trivial TR group as the reference, the mild TR group had a hazard ratio of 2.04 (95% confidence interval 1.09–3.82), while the moderate TR group had a hazard ratio of 5.24 (95% confidence interval 2.31–11.88), showing a significant difference in the incidence of MACE (*Table* *S3*).

In the univariable Cox proportional hazards analysis, the following clinically important parameters were found to be significantly associated with a higher incidence of MACE: mild or greater TR, older age (≥65 years old), LVEF, RVEF and significant (≥mild) ischaemic MR. On the other hand, no significant association was observed for the culprit coronary artery or underlying comorbidities. Multivariable Cox proportional hazards analysis showed that mild or greater TR was independently associated with a higher incidence of MACE (hazard ratio 1.87, 95% confidence interval, 1.01–3.48; *P* = 0.048), even after adjustment for statistically significant clinical variables (*Table* *4*). Older age (≥65 years old) was also a significant predictor of MACE (hazard ratio 2.14, 95% confidence interval, 1.10–4.18; *P* = 0.026).

**Table 4:** Multivariable Cox proportional hazards analysis for major adverse cardiac events

	Univariable		Multivariable	
	HR (95% CI)	*P*	HR (95% CI)	*P*
Mild or greater TR	2.56 (1.48–4.44)	0.001	1.87 (1.01–3.48)	0.048
Older age (≥65 years old)	2.73 (1.46–5.10)	0.002	2.14 (1.10–4.18)	0.026
Male	0.82 (0.44–1.51)	0.521		
STEMI	1.75 (0.75–4.10)	0.196		
Culprit vessel: LAD	1.72 (0.97–3.07)	0.065		
Culprit vessel: RCA	0.70 (0.37–1.33)	0.274		
Hypertension	1.19 (0.67–2.12)	0.549		
Diabetes mellitus	0.92 (0.49–1.72)	0.799		
Dyslipidaemia	0.73 (0.42–1.27)	0.263		
Renal function; eGFR, mL/min/1.73 m^2^	0.99 (0.98–1.00)	0.183		
CKD (eGFR <60 mL/min/1.73 m^2^)	1.55 (0.89–2.69)	0.125		
Paroxysmal atrial fibrillation	1.19 (0.16–8.61)	0.863		
LV end‐diastolic volume, per 1 mL/m^2^	1.02 (0.99–1.04)	0.134		
LV end‐systolic volume, per 1 mL/m^2^	1.03 (1.01–1.06)	0.016		
LV ejection fraction, per 1%	0.96 (0.93–0.98)	0.002	0.97 (0.93–1.00)	0.062
Reduced LVEF (<50%)	1.66 (0.95–2.90)	0.073		
RV end‐diastolic volume, per 1 mL/m^2^	0.99 (0.95–1.02)	0.503		
RV end‐systolic volume, per 1 mL/m^2^	1.02 (0.95–1.09)	0.610		
RV ejection fraction, per 1%	0.96 (0.92–0.99)	0.024	0.97 (0.94–1.01)	0.135
Reduced RVEF (<45%)	0.91 (0.22–3.75)	0.898		
RVLS (free wall), per 1%	0.96 (0.92–1.01)	0.082		
Significant ischaemic MR (≥mild)	1.90 (1.11–3.27)	0.019	1.24 (0.69–2.24)	0.474
Left atrial volume index, per 1 mL/m^2^	1.01 (0.98–1.04)	0.522		
LA dilation (>34 mL/m^2^)	1.38 (0.74–2.58)	0.311		

LV end‐systolic volume was excluded from the multivariable model due to multicollinearity with LV ejection fraction.

CI, confidence interval; CKD, chronic kidney disease; HR, hazard ratio; LA, left atrial; LAD, left anterior descending coronary artery; LV, left ventricular; MR, mitral regurgitation; RCA, right coronary artery; RV, right ventricular; RVLS, right ventricular longitudinal strain; STEMI, ST‐segment elevation myocardial infarction.

### Impact of TR under different conditions

**Figure 3:**
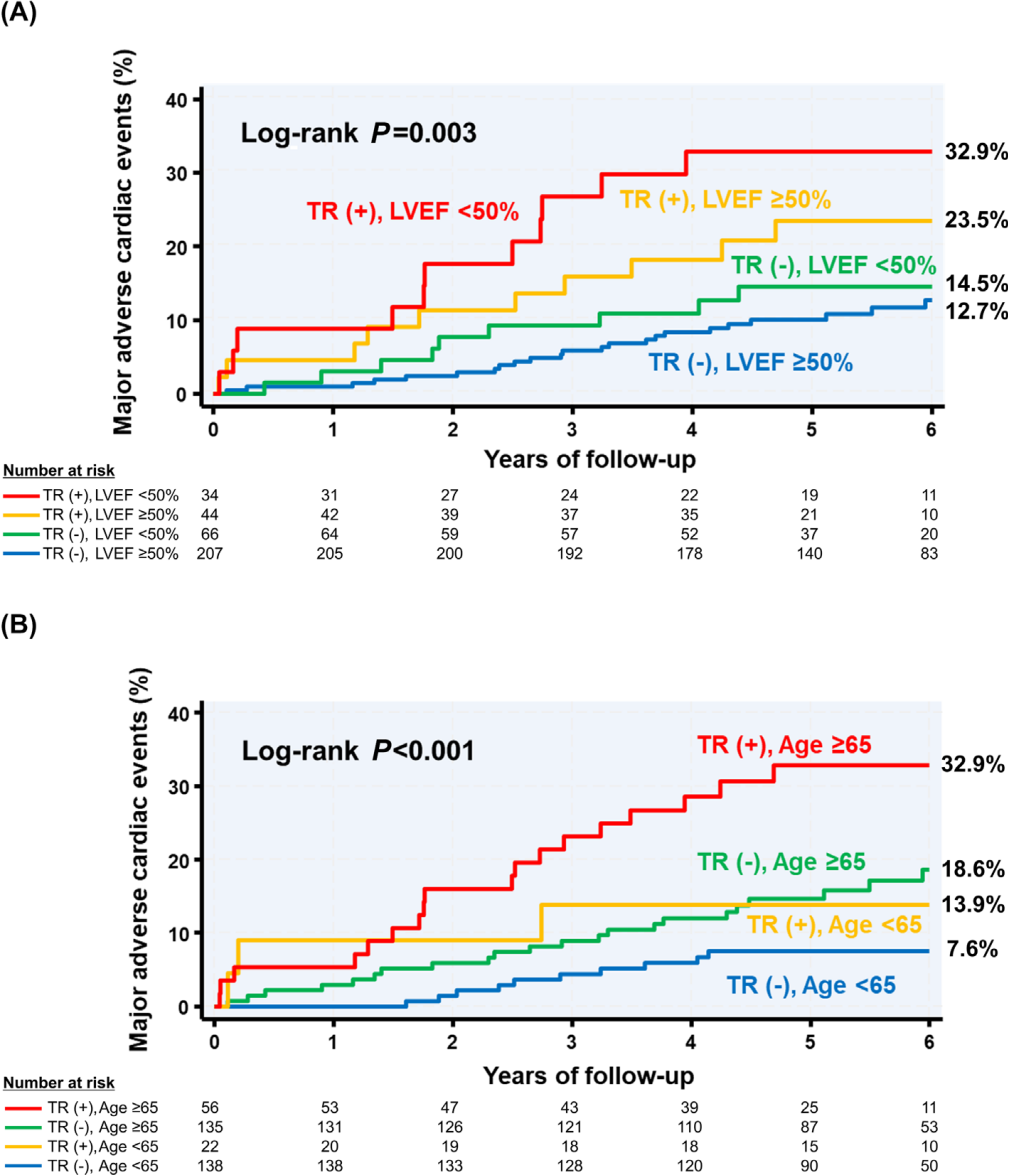
Kaplan–Meier curves stratified by preserved left ventricular ejection fraction with or without mild or greater tricuspid regurgitation showed that the rate of major adverse cardiac events was higher in patients with impaired left ventricular ejection fraction (<50%) and significant (≥mild) tricuspid regurgitation (A). Kaplan–Meier curves stratified by age with or without mild or greater tricuspid regurgitation showed that the rate of major adverse cardiac events was higher in elderly (≥65 years old) patients with mild or greater tricuspid regurgitation (B). LVEF, left ventricular ejection fraction; TR, tricuspid regurgitation.

Even among patients with older age or reduced LVEF, both of which are clinically important conditions, the incidence of MACE was higher in those with mild or greater TR (*Figure* *3* *A,B*).

### Inter‐ and intra‐observer variability of measurements

The inter‐observer coefficients of variation were 5.40% for the average vena contracta width of TR (*r* = 0.98) and 2.38% for LVEF measured by three‐dimensional TTE (*r* = 0.96). The intra‐observer coefficients of variation were 7.55% for the average vena contracta width of TR (*r* = 0.95) and 4.35% for LVEF measured by three‐dimensional TTE (*r* = 0.95).

## Discussion

This study demonstrated four major points. First, patients with AMI complicated by mild or greater TR had a significantly higher incidence of adverse outcomes. Second, the left anterior descending coronary artery as the culprit vessel, left atrial dilation, reduced LVEF and concomitant significant (≥mild) ischaemic MR independently predicted TR in patients with AMI treated with primary PCI. Third, the presence of TR and older age independently predicted poor prognosis in post‐AMI patients. Fourth, among patients with AMI, even mild TR had a significant prognostic impact. This is the first study in which three‐dimensional TTE assessment of cardiac function was used to show that patients with first‐onset AMI treated with appropriate primary PCI demonstrated an association between the presence of mild or greater TR and an increased incidence of adverse post‐AMI cardiovascular events. This suggests a possible prognostic implication of even mild TR in patients with AMI.

There are limited data on the prevalence and prognostic value of TR in patients with AMI who have undergone primary PCI. Few studies have investigated the association between ischaemic heart disease and TR, and most were conducted in populations not undergoing revascularization with primary PCI. As a high‐volume center in cardiovascular care, the study hospital receives emergency patients 24/7, and once a provisional diagnosis of AMI is made in the emergency department, patients are immediately sent to the catheterization laboratory (door‐to‐balloon time was 65 (53–85) minutes). Therefore, our study population received uniform care with standardized reperfusion techniques. The onset‐to‐reperfusion time in this study was 249 (160–485) minutes, and all patients underwent relatively early therapeutic intervention. It would be worthwhile to identify factors that worsen prognosis after AMI, even when appropriate initial treatment for AMI is available.

In this study, we used vena contracta width as the parameter for assessing TR severity due to its feasibility and clinical applicability. Vena contracta width is widely utilized in clinical practice as it is simple, reproducible and practical, particularly in retrospective studies where standardized measurements are essential. To enhance accuracy, we measured vena contracta width in two different echocardiographic views and calculated the average values. This approach aimed to improve the precision of our assessment and reduce potential measurement variability. While effective regurgitant orifice area and regurgitant volume are comprehensive parameters for evaluating TR severity, their use presents certain challenges. These parameters require flow convergence measurements, which can be difficult to obtain accurately due to the complex geometry of the tricuspid valve. Furthermore, in cases of mild TR, the precise quantification is technically challenging and may not provide reliable value. We utilized 3D echocardiography to enhance the accuracy of cardiac function assessment, particularly focusing on RV function, which is known to be challenging to evaluate using 2D echocardiography. Additionally, 3D echocardiography allows for a more precise evaluation of LV function, overcoming the geometric assumptions and foreshortening limitations inherent in 2D echocardiography. The advantages of 3D echocardiography in this study remain significant. Unlike 2D echocardiography, which relies on simplified geometric models, 3D echocardiography provides a more accurate and comprehensive assessment of both LV and RV function. Given the critical role of ventricular function in the prognosis of post‐AMI patients with TR, the use of 3D echocardiography adds clinical value by improving the precision of cardiac performance evaluation.

As TR is associated with various background conditions, including RV or LV dysfunction and pulmonary hypertension, it is often difficult to systematically assess its clinical importance. This study was undertaken purely to investigate the prognostic impact of TR on patients with first‐onset AMI who could be treated with appropriate primary PCI. Patients with other valvular diseases, cardiomyopathies and arrhythmic diseases were excluded because these conditions themselves may significantly impact prognosis. In this study cohort, only a very small number of patients met the criteria for pulmonary hypertension at discharge. As a result, we were unable to evaluate the impact of pulmonary hypertension on TR.

### Prognostic impact of TR

Significant TR is often defined as moderate or severe,^8^, ^9^, ^10^, ^11^, ^13^, ^14^, ^15^, ^29^, ^30^ but one large‐scale study found that even mild TR impacted prognosis.^12^ Mild TR without other conditions is considered benign, but its association with AMI has not been adequately considered. We previously reported that in this era when primary PCI is the standard of care, ischaemic MR was an indicator of adverse outcomes after AMI, even if the regurgitation severity was mild.^7^ Clarifying the adverse prognostic impact of functional regurgitation by both the tricuspid and mitral valves is of great clinical importance in appropriately treated post‐AMI patients. Based on these consideration, this study evaluated whether TR is an independent predictor of worse prognosis in AMI patients, separate from other established prognostic factors after AMI onset, such as age comorbidities, and left ventricular ejection fraction.

The present study showed that in patients with first‐onset AMI who underwent appropriate primary PCI, those with more than mild TR at discharge had a significantly higher incidence of subsequent MACE (*Figure* *2* *A*). A previous meta‐analysis showed that not only moderate or severe TR, but also mild TR, had a significant negative prognostic impact, but the effect of ischaemic heart disease was not examined.^12^ The current study suggests that even mild TR in ischaemic circumstances is a negative prognostic marker (*Figure* *2* *B*, *Table* *S3*), and thus, it follows that more severe TR is as well. Notably, the higher incidence of all‐cause death in the TR (+) group is a significant finding (*Table* *S2*). Although the severity of TR was significantly associated with the incidence of MACE in the univariable analysis, this significance was attenuated in the multivariable model. This attenuation may be partly attributable to the relatively small number of patients in the mild TR group, which may have limited the statistical power of the analysis. Several previous studies conducted in various populations have reported that there was a significant and graded association between the severity of TR and the risk of mortality.^8^, ^9^, ^10^, ^11^, ^12^, ^13^, ^14^ In light of these findings, the presence of TR should be recognized as an important prognostic factor that should be given clinical attention in post‐AMI patients as well. This study aimed to investigate the prognosis of patients following AMI, and while assessing the impact of TR was a key focus, we also included recurrent MI as part of the composite endpoint, as it is known to have a significant impact on the outcomes of patients after AMI. As shown in *Table* *S2*, recurrent MI accounted for only a small proportion of total events (4/53, 7.5%), and its impact on the overall results was minimal. We also conducted an additional analysis excluding recurrent MI from the composite endpoint. The results remained consistent, with TR showing a significant association in the univariable analysis (hazard ratio 2.68, 95% confidence interval 1.51–4.73; *P* = 0.001) and retaining its significance in the multivariable analysis (hazard ratio 2.22, 95% confidence interval 1.25–3.96; *P* = 0.007).

### The prognostic impact of TR in conjunction with other conditions

The present study showed that mild or greater TR may be a poor prognostic marker for patients with AMI, as it was associated with an increased incidence of MACE irrespective of older age (≥65 years old), LVEF, RVEF and ≥mild ischaemic MR (*Table* *4*). The same results were found when grouped by the presence or absence of moderate TR (*Table* *S4*). These results are consistent with a meta‐analysis reported by Wang et al, which found that TR was associated with a poor prognosis independently of RV function.^13^ We showed that patients with mild or greater TR had an even worse prognosis if they also had either reduced LVEF (*Figure* *3* *A*), which itself strongly correlates with post‐AMI prognosis^6^, ^7^ or advanced age (*Figure* *3* *B*). In patients with AMI, there has been an exclusive focus on LV function, LV geometry, ischaemic MR and pulmonary hypertension, but attention should also be paid to TR. The results of this long‐term study suggest that additional follow‐up after acute‐stage treatment is needed to achieve a better prognosis. We speculate that mild TR may become more severe over time and affect prognosis. Continuous, large‐scale observational studies should clarify this hypothesis.

### The relationship between TR and AMI

The exact mechanism of TR development in post‐AMI patients is difficult to determine from this study, as the retrospective design does not allow for ruling out pre‐existing TR before AMI onset. Tricuspid annular dilation and RV dysfunction are the most common causes of functional TR. To eliminate confounding, this study excluded patients with primary TR caused by tricuspid valve prolapse related to tendon or papillary muscle rupture, and those with functional TR due mainly to tricuspid annulus enlargement associated with rhythm disturbances. As previous studies have shown,^31^, ^32^ atrial fibrillation can cause right atrial enlargement and tricuspid annular dilation, contributing to the development and progression of TR. This relationship is particularly relevant in functional TR, where annular dilation plays a key role. In our study, only 6 out of 351 cases (1.7%) had paroxysmal atrial fibrillation, making it difficult to draw definitive conclusions about its association with TR. The study results showed that RV dilation was significantly greater in patients with mild or greater TR than in those without it, but there was no significant difference between the two groups in RV end‐systolic volume or systolic function. These results may indicate that early reperfusion therapy with primary PCI improved RV systolic function at discharge. It cannot be excluded that RV myocardial damage associated with AMI may have caused RV enlargement. However, in patients with mild or greater TR, volume overload also causes RV dilation, but RV systolic function is preserved as a compensatory function. In the present study, RV volume and RV systolic function at discharge were not significant prognostic factors. Patients with mild or greater TR may have a worse prognosis in the chronic phase after hospital discharge due to further RV remodelling and worsening TR. RV infarction can lead to RV systolic dysfunction and RV dilation, both of which have been reported to contribute to the worsening of TR.^33^, ^34^ This study included a subgroup analysis of patients with RV infarction, but it included very few patients and the results were not statistically significant. In addition, RV infarction had already improved in most cases by the time of discharge due to appropriate acute medical intervention. This study did not focus on tricuspid annulus and valve morphology; however, we recognize its potential importance in further understanding the mechanisms and severity of TR. Future studies incorporating a comprehensive 3D echocardiographic analysis of tricuspid valve structure and function will be necessary to expand on these findings. While this study cannot establish a direct causal relationship between AMI and TR, the significantly worse clinical prognosis in AMI patients with mild or greater TR highlights the need for special attention.

### Predictors of mild or greater TR complicating AMI

In the general population, TR is more frequent in the elderly and in women.^12^ In this study, the left anterior descending coronary artery as the culprit vessel, left atrial dilation, impaired LV systolic function (LVEF<50%), and complicated ischaemic MR were independent predictors of TR, while female sex was not. The results of the propensity score matching analysis considering sex differences were also consistent. LV systolic dysfunction and complicated ischaemic MR are possible predictors of TR because they increase pulmonary artery pressure and lead to worsening of TR. While this study evaluated RV volume and systolic function using three‐dimensional echocardiography to increase accuracy, these parameters were not significant predictors of TR after AMI.

### Implications for the treatment of TR

Patients with mild or greater TR after AMI were significantly more likely to be taking medications for heart failure, such as diuretics and mineralocorticoid receptor antagonists, and had higher systolic pulmonary artery pressure. Although mild or greater TR has been reported as a prognostic factor independent of pulmonary hypertension,^13^ treatment of pulmonary hypertension, i.e., more aggressive management of heart failure, can reduce TR and may lower the incidence of MACE. The impact of sodium glucose cotransporter 2 inhibitors, which are now widely used as standard therapy for heart failure, on TR and clinical prognosis is of considerable interest. However, due to the small number of patients receiving these agents in this study, a meaningful evaluation was not feasible. Further research through large‐scale studies is warranted to elucidate their potential effects. While this study focused on TR at discharge after intensive therapeutic intervention, TR may have worsened late in the clinical course, contributing to poor prognosis. Less‐invasive percutaneous tricuspid valve repair and replacement have recently been introduced into clinical practice, and these may significantly impact future guidelines on TR management. This study shows that more than mild TR can worsen prognosis in post‐AMI patients irrespective of culprit vessel. Importantly, it is recommended that comprehensive TR assessment should be systematically assessed with TTE before hospital discharge. We hope that the findings of this study will improve the prognosis of patients with AMI by helping clinicians to manage TR, prevent heart failure worsening, and determine the optimal timing of appropriate direct therapeutic interventions.

## Limitations

We acknowledge several important limitations of our study. First, the number of subjects was relatively small. To accurately assess the clinical impact of TR, which is influenced by a variety of factors, we limited our recruitment to patients with first‐onset AMI who had undergone successful primary PCI, and excluded patients with atrial fibrillation, which can cause tricuspid annular dilation followed by atrial functional TR. In addition, we used three‐dimensional TTE to more accurately assess the cardiac chambers compared to two‐dimensional TTE, but the results cannot be generalized to all AMI patients because we excluded those with atrial fibrillation as it is difficult to perform accurate evaluations under irregular cardiac rhythm. This selection criterion may be a source of selection bias. Second, this single‐center, non‐randomized, observational study was retrospective in nature. The study was subject to selection bias, and thus, the results indicate associations rather than causal relationships. Third, the pre‐existence of TR before infarction cannot be excluded. As our study was retrospective in nature, we could not determine the exact onset of TR in each patient. Therefore, this study did not investigate a causal relationship between TR and AMI but rather focused on the prognostic impact of TR in post‐AMI patients. This limitation is common to many AMI studies. Fourth, quantitative assessment of the severity of TR was not performed. Quantitative evaluation of TR in all patients was not practical, and a simple, easy analysis using the mean vena contracta width seemed reasonable. We hope that the results of this study will be reflected in clinical practice. The next step is to use a larger cohort with more detailed three‐dimensional TTE analysis to determine the pathogenesis of TR. In addition, longer‐term follow‐up is needed to determine how TR improves or worsens after AMI, and its impact on prognosis. Although additional steps are needed to assess this important but complicated issue, we consider the present study to be a first step toward clarifying the clinical evidence of TR in post‐AMI patients.

## Conclusions

The presence of mild or greater TR at discharge may serve as a marker for worse prognosis in post‐AMI patients treated with primary PCI. In addition to considering LVEF and ischaemic MR when managing these patients, it is important to pay more attention to TR, and to manage it appropriately.

## Conflict of interest

None declared.

## Funding

None declared.

## Supporting information


**Table S1.** Medications at discharge.
**Table S2.** Breakdown of MACE events for each group.
**Table S3.** Cox proportional hazards analysis for major adverse cardiac events among the three groups.
**Table S4.** Multivariable Cox proportional hazards analysis for major adverse cardiac events.
**Figure S1.** Patient selection flowchart.
