# Supplementary material for: Clinical impact of tricuspid regurgitation in patients with acute myocardial infarction
Source: ESC Heart Fail. 2025 Jul 14;12(5):3461–74. doi: 10.1002/ehf2.15375 (PMC12450824; doi:10.1002/ehf2.15375)
Supplement: Supplementary file 1 — Table S1. Medications at discharge. Table S2. Breakdown of MACE events for each group. Table S3. Cox proportional hazards analysis for major adverse cardiac events among the three groups. Table S4. Multivariable Cox proportional hazards analysis for major adverse cardiac events. Figure S1. Patient selection flowchart. [file EHF2-12-3461-s001.docx]

**Supplemental Material**

**1. Supplemental Table P2-5**

**2. Supplemental Figure P6**

**1. Supplemental Table**

**Supplemental Table 1.**

**Medications at Discharge**

|  | **Total (n=351)** | **TR (+) (n=78)** | **TR (-) (n=273)** | **p** |
| --- | --- | --- | --- | --- |
| **Beta blocker** | 241 (68.7%) | 56 (71.8%) | 185 (67.8%) | 0.499 |
| **ACE-I or ARB** | 296 (84.3%) | 66 (84.6%) | 230 (84.2%) | 0.937 |
| **MRA** | 20 (5.7%) | 9 (11.5%) | 11 (4.0%) | 0.012 |
| **Loop diuretic** | 51 (14.5%) | 20 (25.6%) | 31 (11.4%) | 0.002 |
| **SGLT2 inhibitors** | 4 (1.1%) | 0 (0.0%) | 4 (1.5%) | 0.364 |

Data are expressed as n (%) of patients.

ACE-I indicates angiotensin-converting enzyme inhibitor; ARB, angiotensin II receptor blocker; MRA, mineralocorticoid receptor antagonist; SGLT2, sodium glucose cotransporter 2; TR, tricuspid regurgitation.

**Supplemental Table 2.**

**Breakdown of MACE Events for Each Group**

|  | **Total (N=351)** | **TR (+) (n=78)** | **TR (-) (n=273)** | **p** |
| --- | --- | --- | --- | --- |
| **MACE** | 53 (15.1%) | 21 (26.9%) | 32 (11.7%) | <0.001 |
| **All-cause death** | 36 (10.3%) | 13 (16.7%) | 23 (8.4%) | 0.034 |
| **Re-hospitalization for HF** | 13 (3.7%) | 7 (9.0%) | 6 (2.2%) | 0.005 |
| **Recurrent MI** | 4 (1.1%) | 1 (1.3%) | 3 (1.1%) | 0.890 |

Data are expressed as n (%) of patients.

TR, indicates tricuspid regurgitation; MACE, major adverse cardiac events; HF, heart failure; MI; myocardial infarction.

**Supplemental Table 3.**

**Cox Proportional Hazards Analysis for Major Adverse Cardiac Events
 Among the Three Groups**

|  | **HR (95% CI)** | **p** | **HR (95% CI)** | **p** |
| --- | --- | --- | --- | --- |
| **Univariable** |  |  |  |  |
| **No, trivial TR** | Reference |  |  |  |
| **Mild TR** | 2.04 (1.09–3.82) | 0.026 | Reference |  |
| **Moderate TR** | 5.24 (2.31–11.88) | <0.001 | 2.57 (1.04–6.37) | 0.042 |
| **Multivariable** |  |  |  |  |
| **No, trivial TR** | Reference |  |  |  |
| **Mild TR** | 1.61 (0.81–3.20) | 0.171 | Reference |  |
| **Moderate TR** | 3.26 (1.24–8.53) | 0.016 | 2.02 (0.75–5.45) | 0.165 |

CI, indicates confidence interval; HR, hazard ratio; TR tricuspid regurgitation. Adjustment variables are older age, left ventricular ejection fraction, right ventricular ejection fraction, and significant ischemic mitral regurgitation.

**Supplemental Table 4.**

**Multivariable Cox Proportional Hazards Analysis for Major Adverse Cardiac Events**

|  | **Univariate** |  | **Multivariable** |  |
| --- | --- | --- | --- | --- |
|  | **HR (95% CI)** | **p** | **HR (95% CI)** | **p** |
| **Moderate TR** | 4.42 (1.99–9.81) | <0.001 | 2.71 (1.08–6.79) | 0.033 |
| **Older age (≥65 years old)** | 2.73 (1.46–5.10) | 0.002 | 2.11 (1.07–4.16) | 0.030 |
| **LV ejection fraction** | 0.96 (0.93–0.98) | 0.002 | 0.97 (0.94–0.99) | 0.038 |
| **Reduced LVEF (<50%)** | 1.66 (0.95–2.90) | 0.073 |  |  |
| **RV ejection fraction** | 0.96 (0.92–0.99) | 0.024 | 0.97 (0.93–1.01) | 0.109 |
| **Reduced RVEF (<45%)** | 0.91 (0.22–3.75) | 0.898 |  |  |
| **Significant ischemic MR (≥mild)** | 1.90 (1.11–3.27) | 0.019 | 1.28 (0.71–2.31) | 0.409 |
| **LA volume index** | 1.01 (0.98–1.04) | 0.522 |  |  |
| **LA dilation (>34 mL/m^2^)** | 1.38 (0.74–2.58) | 0.311 |  |  |

CI, indicates confidence interval; HR, hazard ratio; LA, left atrial; LV, left ventricular; LVEF.

**2. Supplemental Figure with Accompanying Figure Legends**

**Supplemental Figure 1. Patient Selection Flowchart**

**
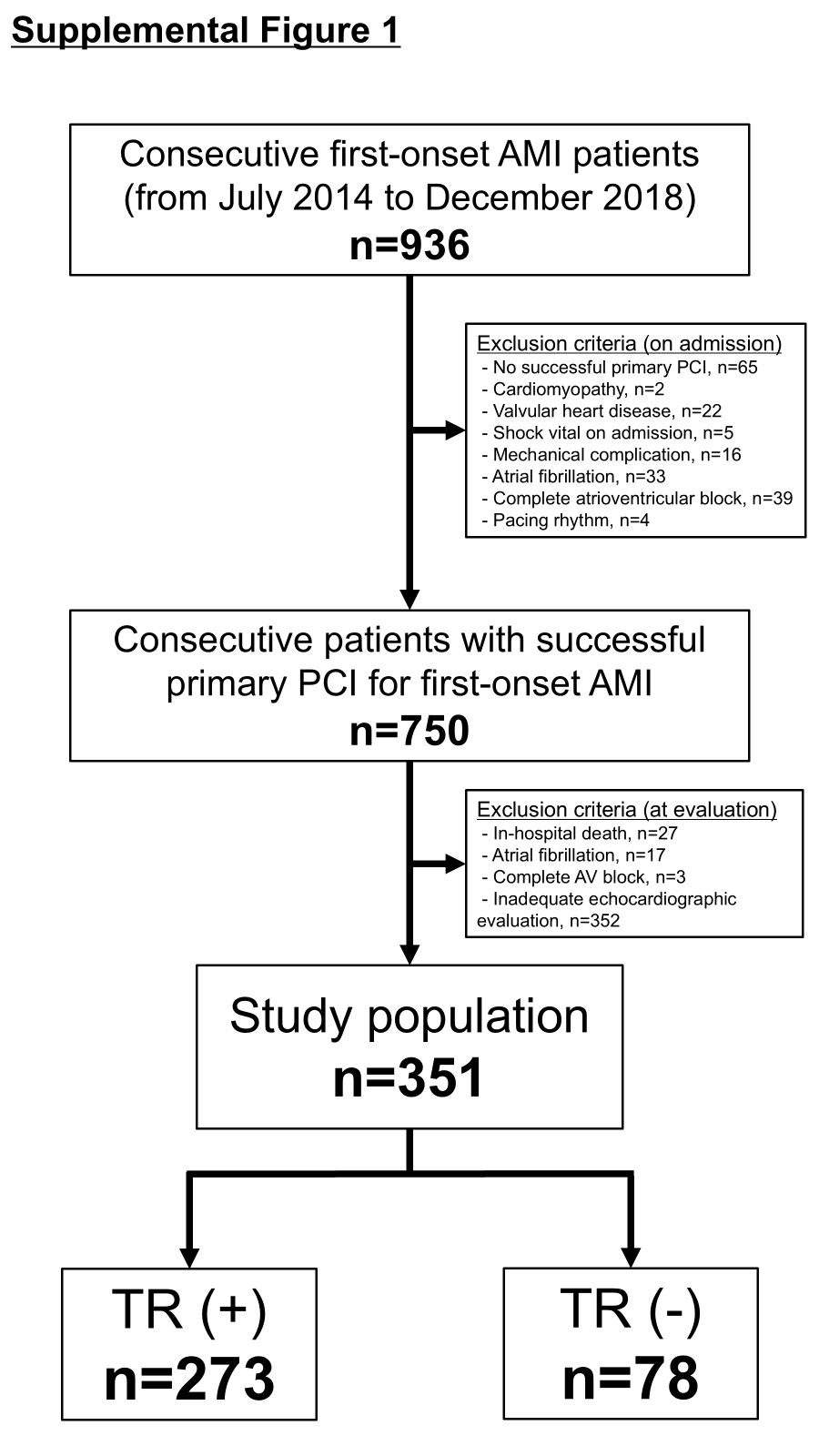
**

AMI indicates acute myocardial infarction; PCI, percutaneous coronary intervention.
